# Does Parenting Perfectionism Ironically Increase Violent Behaviors from Parent towards Children?

**DOI:** 10.3390/children10101704

**Published:** 2023-10-19

**Authors:** Alice Schittek, Isabelle Roskam, Moira Mikolajczak

**Affiliations:** Department of Psychology, UCLouvain, Place Cardinal Mercier 10, 1348 Louvain-la-Neuve, Belgium

**Keywords:** abuse, longitudinal, maltreatment, parental burnout, perfectionistic strivings, perfectionistic concerns, perfectionism, violence

## Abstract

Background: Past research has shown that perfectionistic strivings (PS) and perfectionistic concerns (PC) in the parenting domain are associated with an increase in parental burnout (PB), and that PB causally increases violence towards one’s offspring. One may therefore wonder whether parenting perfectionism may ironically increase violence towards one’s offspring. Objective: To the best of our knowledge, no study has ever investigated whether perfectionism (PS and PC) predicts violence towards one’s offspring, or whether PB could explain this link. In the current pre-registered cross-lagged study, we hypothesized that an increase in PS and PC would lead to an increase in violence via an increase in PB. Method: 228 participants responded to a longitudinal online survey, with three measurement occasions spaced 2 months apart. Results: Contrary to expectations, cross-lagged path models revealed that violence towards the offspring prospectively predicted an increase in PS and PC. Mediation models showed that PB was not a significant mediator. Results of all models did not change when controlling for social desirability. Conclusion: The present study shows that violence towards the offspring increases the risk of PS and PC in parents. Results are discussed in light of the feeling of guilt experienced by parents. Implications: Current worries that parenting perfectionism may paradoxically increase violence appear to be unwarranted at this stage. Moreover, correlation is not causation; thus, emphasizing caution before coming to clinically and societally relevant conclusions in cross-sectional studies. Thus, the PB and child maltreatment literature should slowly shift to using more longitudinal and causal designs.

## 1. Introduction

### 1.1. Theoretical Framework

Over the past decades, parents have been increasingly encouraged to follow guidelines, tips and tricks on how to be a good parent, and even the perfect parent (e.g., [1,2,3]). Parents today have to deal with unrealistic and unattainable ideals of parenthood and parenting (e.g., [4]). They experience an increasing pressure to be perfect. This is because they are conditioned to think that they are the major, if not the sole, determinants of their child’s well-being and future (e.g., [5,6,7]), and because society is, more than ever, setting the terms for what a good parent is and is not (e.g., [8]). As if this is not enough, parents who seek information on how to be the perfect (or a good) parent are now confronted with an enormous amount of sometimes contrasting information (e.g., parents should learn to let go of their children, see [9], versus parents should be involved, see [10]). Thus, the parenting domain has become the breeding ground for opinions and advice on how to be a good parent and how to avoid being a bad parent, thus polarizing parenthood [11,12,13,14].

The foregoing context has led to an ever-increasing number of parents to wish and to try to be as perfect as they can be. Is this a good thing? In other domains, the maladaptive nature of perfectionism has been increasingly documented, to the extent that perfectionism is now considered as a transdiagnostic process in the onset of numerous psychopathologies, such as depression, anxiety, eating disorders, and obsessive compulsive disorder (for a meta-analysis, see [15]). Although the study of deleterious outcomes of perfectionism has widely been investigated in the organizational (for a meta-analysis, see [16]), academic (for meta-analyses, see [17,18]), and sport literature (for a meta-analysis, see [19]), studies have only recently started to venture in the parenthood domain (for a meta-analysis, see [20]). One important and particularly deleterious correlate of parenting perfectionism seems to be parental burnout (e.g., [21,22,23,24]). It is a chronic stress condition manifesting in exhaustion in the parental role, perceiving a contrast with the parent they used to be, feeling fed up with parenthood, and feeling emotionally distant from one’s offspring [25], and has shown to be a global phenomenon [26]. This association has not only been limited to socially prescribed perfectionism [22,23] but also with self-oriented perfectionism (e.g., [21,23]). The latter is particularly interesting because it seems to be paradoxically the most detrimental for the individual and their surroundings (e.g., [27,28,29]), and yet, it is the easiest to be treated (for a meta-analysis, see [30]). Self-oriented perfectionism is characterized by having high standards for the self that are not necessarily required by the environment or the situation [31]. It has two components: perfectionistic strivings and perfectionistic concerns [32,33]. The former is the tendency to have high personal standards and to have a personal quest for perfection, while the latter is the fear of making mistakes and to be negatively perceived by others [33].

Thus, research has shown that both components of self-oriented perfectionism are linked with higher parental burnout. The latter does not only affect the parent, but it also bleeds into the entire family system. Specifically, parental burnout detrimentally affects children [34,35,36,37,38,39], as research has uncovered that it increases violence towards one’s offspring. More particularly, parental burnout has a large (r = 0.49) [40] causal [41] and robust effect on violence towards the offspring (e.g., for correlational, see [39]; for cross-lagged, see [38]; and for experimental, see [41]).

### 1.2. Study Aim and Hypotheses

Considering that parental perfectionism is associated with more parental burnout, and that parental burnout causally leads to more violence towards the offspring (e.g., parents who are burnt out exhibit more worries about personal standards and making mistakes, but also exhibit more maltreating behaviors), is it possible that parents who are trying to be perfect may ironically be at higher risk of being violent towards their offspring? Drawing on a longitudinal cross-lagged design, the present study examines this apparent paradox by testing the hypothesis that trying to be a perfect parent leads to more violence towards one’s offspring. If the hypothesis is confirmed, we further hypothesize that this effect is explained by parental burnout; constantly trying to be a perfect parent will exhaust the parent (increase parental burnout), which in turn will translate into an increase in violent outbursts towards the offspring. Note that although the results may seem obvious given the associations between parenting perfectionism and parental burnout on the one hand (r between 0.23 and 0.37; for a meta-analysis, see [40]) and between parental burnout and violence towards the offspring on the other hand (r = 0.49; for a meta-analysis, see [40]), the results are not necessarily straightforward. Indeed, the link between parental perfectionism and parental burnout has only been demonstrated in correlation studies so far, so they may not hold in longitudinal designs, such as the one adopted in the current study. Identifying whether parental perfectionism is a predictor of violence towards the offspring, and why that is the case (through parental burnout) is paramount in the social context that exhibits an increasing pressure on parents towards perfection.

Four hypotheses were posited:

**Hypothesis** **1:** *Perfectionistic strivings prospectively predicts violence towards one’s offspring;*

**Hypothesis** **2:** *Perfectionistic concerns prospectively predict violence towards one’s offspring;*

**Hypothesis** **3:** *Perfectionistic strivings (at time 1) leads to more parental burnout (at time 2), which in turn leads to more violence towards one’s offspring (at time 3)*;

**Hypothesis** **4:** *Perfectionistic concerns (at time 1) leads to more parental burnout (at time 2), which in turn leads to more violence towards one’s offspring (at time 3)*.

## 2. Materials and Methods

### 2.1. Procedure

The current study has received approval by the Ethics Committee, and it is part of a larger project on the relation between parental burnout and violence. A priori sample size estimations were performed using the N:q rule proposed by [42] and supported by [43], according to which path models should have between 10 to 20 participants (N) for every parameter that requires statistical estimates. Participants were recruited on Prolific (https://www.prolific.ac), which is a participant recruitment website based in Cambridge (United Kingdom). Prolific bridges the gap between researchers and reliable participants, and it is now widely used around the world by renowned universities. It offers fast and reliable data for research purposes. Researchers can enter their research proposal and select screening criteria for specific participants to participate (e.g., only participants who have children). The sampling type consisted of simple random sampling, in which parents on Prolific all had an equal chance to participate in the study. In order to avoid self-selection bias, participants were informed that the study was on parenthood in general. Considering that the current study is part of a larger study, and that the recruitment was restricted by economic constraints, the first measurement occasion recruited 1000 participants, whereas time 2 and 3 recruited 300. Data collection happened between June 2022 and January 2023, in which the three waves of questionnaires were spaced 2 months apart (June 2022, August 2022, and October 2022).

### 2.2. Measures

Following the signature of informed consent, the following sociodemographic variables were collected: age, gender, number of children under the same roof, number of children they have in total, age of the youngest child still living at home, age of the oldest child, parent’s educational level, working regimen, and net monthly household income.

Parental perfectionism was measured using the Brief Parenting Perfectionism Scale (adapted by [44], based on prior perfectionism scales of [21,45]). It is a 6-item questionnaire in which participants answer on a 5-point scale using the following options: does not fit me at all (1) to fits me perfectly (5). The measure encompasses two factors: perfectionistic strivings (time 1: α = 0.75, ω = 0.76; time 2: α = 0.77, ω = 0.78; time 3: α = 0.78, ω = 0.79). These reflect the very high standards regarding oneself as parent (e.g., “I aim to be a perfect parent”) and perfectionistic concerns (time 1: α = 0.77, ω = 0.80; time 2: α = 0.74, ω = 0.76; time 3: α = 0.73, ω = 0.76), which reflects a tendency to be self-critical and to be concerned over mistakes in one’s parenting role (e.g., “As a parent, it’s awful to fail in front of others”) (for more details, see [44]). Scores are computed by summing the answer to each item. Higher scores reflect higher perfectionistic tendencies.

Parental burnout was measured using the Parental Burnout Assessment (PBA; time 1: α = 0.96, ω = 0.96; time 2: α = 0.96, ω = 0.96; time 3: α = 0.96, ω = 0.97). The PBA is a 23-item questionnaire [24,26] in which parents rate each item/symptom on a 7-point frequency scale using the following options: never (0), a few times a year, once a month or less, a few times a month, once a week, a few times a week, and every day (6). The items form four factors related to the parental role: exhaustion (e.g., “I’m so tired out by my role as a parent that sleeping doesn’t seem like enough”), contrast (e.g., “I don’t think I’m the good father/mother that I used to be to my child(ren)”), feeling fed up (e.g., “I can’t stand my role as a father/mother anymore”), and emotional distancing (e.g., “Outside the usual routines (lifts in the car, bedtime, meals), I’m no longer able to make an effort for my child(ren)”). The parental burnout score is calculated by summing the answer to each item. Higher scores reflect a higher level of parental burnout.

Violence towards offspring was assessed using the Parental Violence Scale ([37]) (time 1: α = 0.70, ω = 0.75; time 2: α = 0.68, ω = 0.75; time 3: α = 0.67, ω = 0.75), which is a 15-item self-report questionnaire. Participants respond to each item on a frequency scale using the following scheme: never (1), less than once a month, about once a month, a few times a month, once a week, several times a week, every day, and several times a day (8). The scale measures verbal (e.g., “I sometimes say things to my child that I then regret (threats, insults, use of silly nicknames etc.)”), physical (e.g., “When I am angry, I sometimes throw things at my child”), and psychological violence (e.g., “I sometimes threaten to abandon my child if s/he is not good”). The violence score is calculated by summing the answer to each item. Higher scores reflect a higher presence of violent behaviors.

Considering that violence towards the offspring is quite a delicate and taboo subject, social desirability was measured as a control variable (time 1: α = 0.72, ω = 0.72; time 2: α = 0.72, ω = 0.73; time 3: α = 0.73, ω = 0.74). Social desirability was measured using the Marlowe–Crowne Social Desirability Scale—Short Form (based on [46]; adapted by [47]), which consists of thirteen items for which participants have to indicate whether they agree or disagree by selecting either “true”’ (1) or “false” (0) (e.g., “It is sometimes hard for me to go on with my work if I am not encouraged”). The total score is computed by summing the answers to the items. The scale is unifactorial, and higher scores reflect a higher tendency to change responses to match to what is socially accepted or desirable.

Since these questionnaires were part of a lengthier study, three attention-check items (e.g., “In the following question, select the number between three and five”) were randomly inserted in the survey to make sure that participants did not respond randomly or inattentively.

### 2.3. Exclusion Criteria

Participants who did not meet the following criteria were excluded from all analyses: participants who were underage (under 18 years of age), participants who incorrectly answered to at least one attention check, participants who did not answer to all the variables under study, and participants who did not respond to all three measurement occasions.

### 2.4. Participants

At the beginning of the recruitment process, 1000 people were invited to participate to the study. Due to economical constraints, only 300 of them were then randomly invited to answer to the second and third measurement occasion. At time 1, a total of 914 participants responded, but only 794 met the inclusion criteria. At time 2, a total of 259 participated, and only 249 were eligible, while 10 were excluded due to answering incorrectly to attention checks. At time 3, a total of 241 participants responded to the study, but only 236 met the prior-mentioned criteria, and exclusions were due to not answering correctly to the attention checks. Only participants who answered the three measurement occasions were kept for analyses. Three participants were then excluded for not answering to all the variables, rending the sample to 233 participants. After excluding five multivariate outliers, the final sample consisted of 228 participants across three measurement occasions. Analyses were run with and without multivariate outliers. The final sample had mostly women (65.8%), on average being 37.8 years old (SD = 9.70) mostly living with one (46.9%) or two (34.2%) children, with one (40.4%) or two (36.4%) children in total. The youngest child was aged mostly between two (12.7%) and three (8.8%) years of age, and the oldest child was mostly two (6.1%), three (6.1%), five (6.1%), or ten (5.3%) years old. The majority of participants completed an undergraduate education (48.7%), worked full time (69.7%), earned between USD 2000 and 2745 monthly, and were in a biparental family (73.2%).

### 2.5. Statistical Analyses

Assumptions for correlations and cross-lagged path analyses were checked before carrying on with the analyses. Violations in normality were detected and dealt with appropriately (e.g., Kendall’s τ for non-normal correlations, see [48]). Correlation analyses were carried out using Jamovi’s “Regression” package [49], and cross-lagged analyses were carried out using the open software R Version 2023.03.0+386 [50] with lavaan’s package [51]. Mediation cross-lagged path analyses were previously pre-registered on Open Science Framework before data analysis (available at: https://osf.io/dx3m7/?view_only=66b51fab0c28418d949c5cf349e33c4c (posted on 25 April 2023). Correlations were then performed to understand the bivariate link between variables, independently of other variables.

Prior to interpreting cross-lagged models, fit indices were estimated and reviewed. Considering the particular sample-size sensitivity of the value of χ^2^ and the Root Mean Square Error of Approximation (RMSEA) [43,52], we considered that models showed adequate fit when at least two of the less sensitive indices (i.e., CFI, SRMR, TLI) met the aforementioned criteria: The Comparative Fit Index (CFI) was above 0.90, the Standardized Root Mean Square Residual (SRMR) was below 0.08 [43], and when the Tucker–Lewis Index (TLI) was above 0.90 [53]. χ^2^ and RMSEA were reported as they are still one of the most reported indices in path analysis. χ^2^ must ideally be non-significant, and RMSEA, below 0.08.

We then estimated four full cross-lagged models. The first and second model tested the longitudinal direct effect of perfectionistic strivings (model 1) and perfectionistic concerns (model 2) on violence towards one’s offspring, in three-measurement occasions models. Then, two additional cross-lagged models were computed to test the longitudinal mediating effect of parental burnout in the link between perfectionistic strivings and violence (model 3), and in the link between perfectionistic concerns and violence (model 4). For all four models, first, Mardia’s test was performed to assess the multivariate normal distribution of endogenous variables.

## 3. Results

Mardia’s test was performed and indicated that this assumption of multivariate normality was violated in all four models [54,55] (model 1: for skewness: χ^2^(20) = 263, *p* < 0.001; for kurtosis: z = 15.2, *p* < 0.001; model 2: for skewness: χ^2^(20) = 291, *p* < 0.001; for kurtosis: z = 14.6, *p* < 0.001; model 3: skewness: χ^2^(56) = 497, *p* < 0.001, kurtosis: z = 17.7, *p* < 0.001; and model 4: for skewness: χ^2^(56) = 530, *p* < 0.001, for kurtosis: z = 18.4, *p* < 0.001). Cross-lag path analyses were therefore estimated using more robust methods, such as the Bollen–Stine bootstrapping method [43,56].

Correlations between variables at each time point can be viewed in Table 1. Kendall’s τ was preferred over Pearson’s r, considering the non-normal (but expected) data distribution. Correlations were consistent across the three time points. Perfectionistic strivings and perfectionistic concerns were both positively correlated with violence towards one’s offspring across time points. Parental burnout was positively correlated with violence. Only perfectionistic concerns were positively correlated with parental burnout. On the contrary, perfectionistic strivings did not correlate with parental burnout at any time point. These results held with and without multivariate outliers.

**Figure 1:**
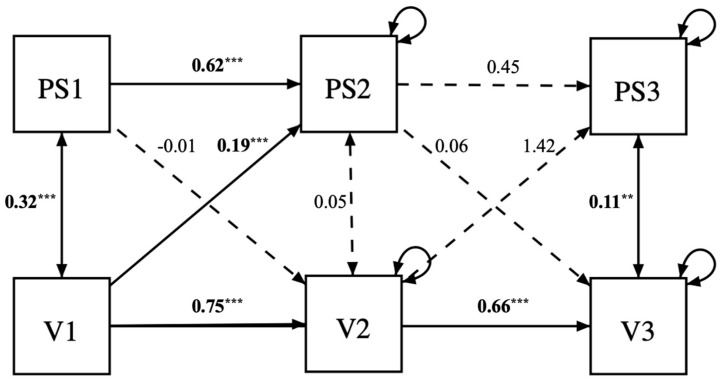
Cross-lagged model investigating the direct effect of perfectionistic strivings on violence towards one’s offspring. Note: PS1 = perfectionistic strivings at time 1; PS2 = perfectionistic strivings at time 2; PS3 = perfectionistic strivings at time 3; V1 = violence at time 1; V2 = violence at time 2; V3 = violence at time 3. Significant paths and covariances are in black, and their standardized coefficients are marked in bold with asterisks (** *p* < 0.01, *** *p* < 0.001). Non-significant paths and covariances are depicted with dotted lines, and their coefficients are not in bold and with no asterisks. Bootstrapping = 1000 samples.

The first model investigating the longitudinal link between perfectionistic strivings and violence showed adequate fit (χ^2^(2) = 50.47, *p* < 0.001; CFI = 0.93; TLI = 0.50; RMSEA = 0.32, 95% CI = [0.25, 0.41], SRMR = 0.04). However, contrary to our hypotheses, perfectionistic strivings did not prospectively predict violence. In fact, it was violence at time 1 that predicted perfectionistic strivings at time 2 (β = 0.19, *p* < 0.001). The remaining paths were non-significant, such as perfectionistic strivings at time 1 predicting violence at time 2 (β = −0.01, *p* = 0.91), perfectionistic strivings at time 2 predicting violence at time 3 (β = 0.06, *p* = 0.23), and violence at time 2 predicting perfectionistic strivings at time 3 (β = 1.42, *p* = 0.39) (Figure 1). The results did not change after controlling for social desirability. Analyses were re-performed with multivariate outliers, and the results did not change except for the path between violence at time 2 predicting perfectionistic strivings at time 3 that became marginally significant (β = 1.23, *p* = 0.04)

**Figure 2:**
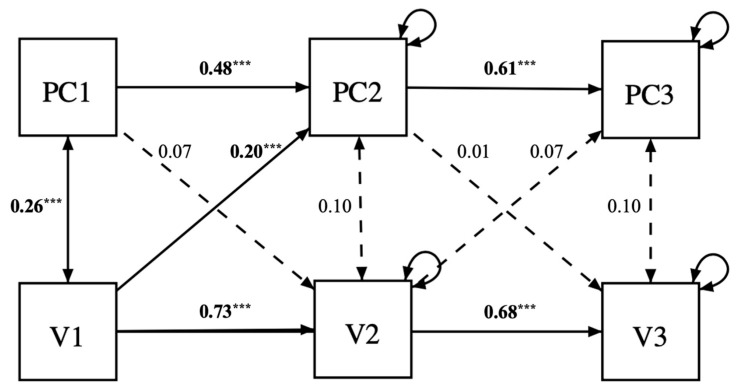
Cross-lagged model investigating the direct effect of perfectionistic concerns on violence towards one’s offspring. *Note:* PC1 = perfectionistic concerns at time 1; PC2 = perfectionistic concerns at time 2; PC3 = perfectionistic concerns at time 3; V1 = violence at time 1; V2 = violence at time 2; V3 = violence at time 3. Significant paths and covariances are in black, and their standardized coefficients are marked in bold with asterisks (*** *p* < 0.001). Non-significant paths and covariances are depicted with dotted lines, and their coefficients are not in bold and with no asterisks. Bootstrapping = 1000 samples.

For the second model estimating the cross-lagged effect of perfectionistic concerns on violence towards the offspring, it showed adequate fit (χ^2^(4) = 80.5, *p* < 0.001; CFI = 0.87; TLI = 0.53; RMSEA = 0.29, 95% CI = [0.24, 0.34], SRMR = 0.06). Contrary to our expectations, perfectionistic concerns did not prospectively predict violence. In fact, it was violence at time 1 that predicted perfectionistic concerns at time 2 (β = 0.2, *p* < 0.001). The remaining paths were not significant: perfectionistic concerns at time 1 predicting violence at time 2 (β = 0.07, *p* = 0.21), perfectionistic concerns at time 2 predicting violence at time 3 (β = 0.001, *p* = 0.90), and perfectionistic concerns at time 3 being predicted by violence at time 2 (β = 0.07, *p* = 0.22) (Figure 2). The results did not change after controlling for social desirability nor for the presence of multivariate outliers.

Considering that the mediation hypotheses relied on the corroboration of Hypothesis 1 or 2, and that neither were supported, mediation analyses between perfectionism strivings and concerns, parental burnout, and violence appear to be unwarranted. However, in a context where the criteria to test mediations have become more liberal [57], we performed them, nevertheless. As expected, none of these mediations were significant (see Figures S1 and S2 in Supplemental Materials).

## 4. Discussion

The present study shows that it is not parenting perfectionism that drives violence, but rather it is the other way around. Showing that the direction of the association is the opposite of what was originally expected is a big step forward in the link between perfectionism and violence, as past studies solely used cross-sectional methods (e.g., [27,28,29]). Our results do not support the fear that parental perfectionism would feed parental violence (through the mediation of parental burnout). Instead, our findings suggest that being violent against one’s children is prospectively associated with a raise in both parents’ standards and concerns about making mistakes as parents. One may obviously wonder whether these reflect “true” effects or are mere socially desirable responses. Interestingly, the standardized betas for the effect of violence on prospective perfectionisms did not change after controlling for social desirability, suggesting that the above-mentioned effects are not merely due to social desirability.

The fact that parent-reported violence towards their children is associated with a prospectively higher levels of parenting perfectionism may be explained by guilt. Imagine a parent who has been violent with their children, which then sparks a sense of guilt in them. To reduce or deal with this sense of guilt, the parent could aim to be a better parent, not only to preserve their self-esteem but also for the children’s sake. This reconciliation attempt could thus result in an increase in the parent’s standards (perfectionistic strivings) and in the concern of being perfect (perfectionistic concerns). Past cross-sectional studies have shown that violence is negatively related to guilt [58,59,60], and that guilt is then related with an increase in perfectionism [61,62]. Considering that the current interpretation is based on correlations and that correlation is not causation (as also shown in the present study), future studies should test the relationship between variables in (i) a mediation model to see whether guilt explains why violence towards the offspring leads to more parental perfectionism, and (ii) in a moderation model to see in what case violence leads to more parental perfectionism (i.e., when the parent feels guilty), making use of designs that allow for the indication of causality.

The current study also suggests that, although violence may increase parenting perfectionism, the latter does not seem to reduce violent behaviors in subsequent times. Two interpretations can explain this. First, it is possible that the methodology employed in the current study does not allow for the visualization of the effects of perfectionism on violence because the time lapse is too short. In fact, to the author’s knowledge, it is currently unknown if and after how much time an increase in parenting perfectionism could reduce violent behaviors. This could explain why we observed a link between violence at time 1 and perfectionism (strivings and concerns) at time 2, but no link between violence at time 2 and perfectionism (strivings and concerns) at time 3. More longitudinal studies on parenting perfectionism and violence with different time lapses should be conducted to better understand the relationship that is present. Second, if in fact the time lapses in the current study are not an issue, another explanation is that parents, after having been violent, have an increase in their standards and concerns over being a good parent, but that this subsequent perfectionism is not enough of a driving force to later decrease violence. If this is the case, this finding has a strong clinical implication, underlying that violent behaviors exceed the want to be a better parent. Thus, understanding why wanting to be a better parent does not seem to translate into a decrease in violence is of utmost importance. Considering the clinical implication, this should potentially be investigated in more qualitative or mixed-methods studies, as a potential explanation would emerge from the parent’s words, thereby allowing for more quantitative investigations. Aside from research, this finding should also be considered by clinicians, who should try to understand what leads to violence for each parent, even when they are concerned over being perfect parents.

Another important finding of the present study is that, as pointed above, our results do not seem to support the fact that parental perfectionism would feed parental violence through the mediation of parental burnout. The mediation hypotheses were built upon highly and consistent correlations between parental burnout and perfectionism. Yet, most parental burnout research is still cross-sectional (90% according to Mikolajczak et al.’s meta-analysis [40]). Although research has unraveled important links between this syndrome and numerous variables, correlation does not mean causation. It is therefore very important to probe the causality and direction of cross-sectional associations. For example, the link between parental burnout and perfectionism has only ever been investigated cross-sectionally [21,22,23,44], showing consistent associations between perfectionism and parental burnout. The cross-sectional nature of studies can lead to false and dangerous causal conclusions. In fact, had we performed a mediation model with only time 1 in the current study, we would have concluded that parental burnout significantly mediates the link between perfectionistic concerns and violence towards the offspring (results can be viewed in Supplementary Table S1). Whereas longitudinally, this does not hold. Our results emphasize the urgent need for the field to shift to more causal or quasi-causal experimental designs to understand if variables entertain a causal relation with parental burnout. Considering the sensitive nature of parental burnout and violence against one’s offspring and their impact on society, we have much to gain in using research designs that would allow for more robust interpretations.

### 4.1. Limitations

Despite its strengths, the limitations of the current study should also be acknowledged. First, although the sample seems to be demographically well balanced, it is not the case for the family type. Most parents in the current study are in a biparental family. This does not allow us to generalize results to single parents, who are more at risk of child maltreatment (for a meta-analysis, see [63]). Second, a further limitation is the arbitrary choice of time intervals between measures (2 months). Although this time lag appears sound to detect changes in the variables under study, the present results would benefit from being replicated in intensive longitudinal methods (with measures every day) to detect more dynamic changes between variables.

### 4.2. Implication

The current findings have implications in future research, which should try to understand why violence may increase perfectionism, through more causally indicative research designs. Clinicians should also try to understand why parents resort to violence, and how it relates to worries in perfectionism. Ideally, both the research and the clinical fields should collaborate in order to elucidate why and in what case there is a link between violence and perfectionism.

### 4.3. Conclusions

In conclusion, violence towards one’s offspring is prospectively linked to an increase in perfectionistic strivings and perfectionistic concerns in parents, which unfortunately does not seem to prospectively translate in less violence (at least in the short term). Future studies should rely on (quasi-)causal designs to unravel the effects of child maltreatment on the whole family system.

## Figures and Tables

**Table 1:** Descriptive statistics and Kendall’s τ correlations for study variables.

Bivariate Correlations	*n*	Time 1	Time 2	Time 3
Perfectionistic Strivings and Violence	228	0.24 ***	0.24 ***	0.25 ***
Perfectionistic Concerns and Violence	228	0.19 ***	0.23 ***	0.15 **
Parental Burnout and Violence	228	0.27 ***	0.29 ***	0.27 ***
Perfectionistic Strivings and Parental Burnout	228	0.01	0.01	−0.03
Perfectionistic Concerns and Parental Burnout	228	0.16 ***	0.13 ***	0.15 ***

Note. Parental burnout at time 1 (*M* = 30.7, *SD* = 27.1), time 2 (*M* = 26.1, *SD* = 24.8), and time 3 (*M* = 25.4, *SD* = 25.6). Violence at time 1 (*M* = 22.3, *SD* = 6.68), time 2 (*M* = 21.2, *SD* = 5.73), and time 3 (*M* = 20.5, *SD* = 5.29). Perfectionistic strivings at time 1 (*M* = 7.77, *SD* = 3.03), time 2 (*M* = 7.68, *SD* = 3.08), and time 3 (*M* = 7.68, *SD* = 3.08). Perfectionistic concerns at time 1 (*M* = 6.21, *SD* = 2.88), time 2 (*M* = 6.04, *SD* = 2.70), and time 3 (*M* = 5.98, *SD* = 2.65). ** *p* < 0.01, *** *p* < 0.001.

## Data Availability

Data can be accessed by contacting the authors of the manuscript.

## Supplementary Materials

The following supporting information can be viewed at: https://www.mdpi.com/article/10.3390/children10101704/s1.

## References

[1] Lapin C. (2020). *Parenting in the Age of Perfection: A Modern Guide to Nurturing a Success Mindset*; Tutor La. https://www.amazon.com/PARENTING-AGE-PERFECTION-Nurturing-Success-ebook/dp/B08468Q7LW.

[2] Pantley E. (2009). Perfect Parenting: The Dictionary of 1000 Parenting Tips.

[3] Roberts T. (2022). Parenting towards Perfection: The Insights of a Christian Mom.

[4] Nomaguchi K., Milkie M.A. (2020). Parenthood and Well-Being: A Decade in Review. J. Marriage Fam..

[5] Adams E.A. (2020). Intensive Parenting Ideologies and Risks for Recidivism among Justice-Involved Mothers. Women Crim. Justice.

[6] Lind J., Westerling A., Sparrman A., Dannesboe K.I., Sparrman A., Westerling A., Lind J., Dannesboe K.I. (2016). Introduction: Doing Good Parenthood. Doing Good Parenthood: Ideals and Practices of Parental Involvement.

[7] Rizzo K.M., Schiffrin H.H., Liss M. (2013). Insight into the Parenthood Paradox: Mental Health Outcomes of Intensive Mothering. J. Child Fam. Stud..

[8] Sperling J.H. (2013). Reframing the Work-Family Conflict Debate by Rejecting the Ideal Parent Norm. J. Gend. Soc. Policy Law.

[9] Obradović J., Sulik M.J., Shaffer A. (2021). Learning to Let Go: Parental over-Engagement Predicts Poorer Self-Regulation in Kindergartners. J. Fam. Psychol..

[10] Tan C.Y., Lyu M., Peng B. (2020). Academic Benefits from Parental Involvement Are Stratified by Parental Socioeconomic Status: A Meta-Analysis. Parenting.

[11] DeVore E.R., Ginsburg K.R. (2005). The Protective Effects of Good Parenting on Adolescents. Curr. Opin. Pediatr..

[12] Eve P.M., Byrne M.K., Gagliardi C.R. (2014). What Is Good Parenting? The Perspectives of Different Professionals. Fam. Court Rev..

[13] Smith M. (2010). Good Parenting: Making a Difference. Early Hum. Dev..

[14] Unnever J.D., Cullen F.T., Agnew R. (2006). Why Is “Bad” Parenting Criminogenic? Implications From Rival Theories. Youth Violence Juv. Justice.

[15] Limburg K., Watson H.J., Hagger M.S., Egan S.J. (2017). The Relationship Between Perfectionism and Psychopathology: A Meta-Analysis. J. Clin. Psychol..

[16] Harari D., Swider B.W., Steed L.B., Breidenthal A.P. (2018). Is Perfect Good? A Meta-Analysis of Perfectionism in the Workplace. J. Appl. Psychol..

[17] Madigan D.J. (2019). A Meta-Analysis of Perfectionism and Academic Achievement. Educ. Psychol. Rev..

[18] Osenk I., Williamson P., Wade T.D. (2020). Does Perfectionism or Pursuit of Excellence Contribute to Successful Learning? A Meta-Analytic Review. Psychol. Assess..

[19] Hill A.P., Mallinson-Howard S.H., Jowett G.E. (2018). Multidimensional Perfectionism in Sport: A Meta-Analytical Review. Sport Exerc. Perform. Psychol..

[20] Evans C., Kreppner J., Lawrence P.J. (2022). The Association between Maternal Perinatal Mental Health and Perfectionism: A Systematic Review and Meta-Analysis. Br. J. Clin. Psychol..

[21] Kawamoto T., Furutani K., Alimardani M. (2018). Preliminary Validation of Japanese Version of the Parental Burnout Inventory and Its Relationship with Perfectionism. Front. Psychol..

[22] Raudasoja M., Sorkkila M., Aunola K. (2022). Self-Esteem, Socially Prescribed Perfectionism, and Parental Burnout. J. Child Fam. Stud..

[23] Sorkkila M., Aunola K. (2019). Risk Factors for Parental Burnout among Finnish Parents: The Role of Socially Prescribed Perfectionism. J. Child Fam. Stud..

[24] Lin G.-X., Szczygieł D., Piotrowski K. (2022). Child-Oriented Perfectionism and Parental Burnout: The Moderating Role of Parents’ Emotional Intelligence. Personal. Individ. Differ..

[25] Roskam I., Brianda M.E., Mikolajczak M. (2018). A Step Forward in the Conceptualization and Measurement of Parental Burnout: The Parental Burnout Assessment (PBA). Front. Psychol..

[26] Roskam I., Aguiar J., Akgun E., Arikan G., Artavia M., Avalosse H., Aunola K., Bader M., Bahati C., Barham E.J. (2021). Parental Burnout Around the Globe: A 42-Country Study. Affect. Sci..

[27] Aparicio-Flores M.P., Vicent M., Freire-Andino R.O., Sanmartín R., Gonzálvez C., García-Fernández J.M. (2022). Profiles of Perfectionistic Automatic Thoughts and Aggression. Psychol. Rep..

[28] Chester D.S., Merwin L.M., DeWall C.N. (2015). Maladaptive Perfectionism’s Link to Aggression and Self-Harm: Emotion Regulation as a Mechanism. Aggress. Behav..

[29] Stoeber J., Noland A.B., Mawenu T.W.N., Henderson T.M., Kent D.N.P. (2017). Perfectionism, Social Disconnection, and Interpersonal Hostility: Not All Perfectionists Don’t Play Nicely with Others. Personal. Individ. Differ..

[30] Lloyd S., Schmidt U., Khondoker M., Tchanturia K. (2015). Can Psychological Interventions Reduce Perfectionism? A Systematic Review and Meta-Analysis. Behav. Cogn. Psychother..

[31] Shafran R., Mansell W. (2001). Perfectionism and Psychopathology: A Review of Research and Treatment. Clin. Psychol. Rev..

[32] Stoeber J., Gaudreau P. (2017). The Advantages of Partialling Perfectionistic Strivings and Perfectionistic Concerns: Critical Issues and Recommendations. Personal. Individ. Differ..

[33] Stoeber J., Otto K. (2006). Positive Conceptions of Perfectionism: Approaches, Evidence, Challenges. Personal. Soc. Psychol. Rev..

[34] Chen B.-B., Qu Y., Yang B., Chen X. (2022). Chinese Mothers’ Parental Burnout and Adolescents’ Internalizing and Externalizing Problems: The Mediating Role of Maternal Hostility. Dev. Psychol..

[35] Griffith A.K. (2022). Parental Burnout and Child Maltreatment During the COVID-19 Pandemic. J. Fam. Violence.

[36] Hansotte L., Nguyen N., Roskam I., Stinglhamber F., Mikolajczak M. (2021). Are All Burned Out Parents Neglectful and Violent? A Latent Profile Analysis. J. Child Fam. Stud..

[37] Mikolajczak M., Brianda M.E., Avalosse H., Roskam I. (2018). Consequences of Parental Burnout: Its Specific Effect on Child Neglect and Violence. Child Abus. Negl..

[38] Mikolajczak M., Gross J.J., Roskam I. (2019). Parental Burnout: What Is It, and Why Does It Matter?. Clin. Psychol. Sci..

[39] Szczygieł D., Sekulowicz M., Kwiatkowski P., Roskam I., Mikolajczak M. (2020). Validation of the Polish Version of the Parental Burnout Assessment (PBA). New Dir. Child Adolesc. Dev..

[40] Mikolajczak M., Aunola K., Sorkkila M., Roskam I. (2023). 15 Years of Parental Burnout Research: Systematic Review and Agenda. Curr. Dir. Psychol. Sci..

[41] Brianda M.E., Roskam I., Gross J.J., Franssen A., Kapala F., Gérard F., Mikolajczak M. (2020). Treating Parental Burnout: Impact of Two Treatment Modalities on Burnout Symptoms, Emotions, Hair Cortisol, and Parental Neglect and Violence. Psychother. Psychosom..

[42] Jackson D.L. (2003). Revisiting Sample Size and Number of Parameter Estimates: Some Support for the N:Q Hypothesis. Struct. Equ. Model. A Multidiscip. J..

[43] Kline R.B. (2016). Principles and Practice of Structural Equation Modeling.

[44] Lin G.-X., Szczygieł D., Hansotte L., Roskam I., Mikolajczak M. (2021). Aiming to Be Perfect Parents Increases the Risk of Parental Burnout, but Emotional Competence Mitigates It. Curr. Psychol..

[45] Snell W.E., Overbey G.A., Brewer A.L. (2005). Parenting Perfectionism and the Parenting Role. Personal. Individ. Differ..

[46] Crowne D.P., Marlowe D. (1960). A New Scale of Social Desirability Independent of Psychopathology. J. Consult. Psychol..

[47] Reynolds W.M. (1982). Development of Reliable and Valid Short Forms of the Marlowe-Crowne Social Desirability Scale. J. Clin. Psychol..

[48] Long J.D., Cliff N. (1997). Confidence Intervals for Kendall’s Tau. Br. J. Math. Stat. Psychol..

[49] The Jamovi Project Jamovi, Version 1.6; 2021. www.jamovi.org.

[50] RStudio Team (2020). RStudio: Integrated Development for R. RStudio.

[51] Rosseel Y. (2012). Lavaan: An R Package for Structural Equation Modeling. J. Stat. Soft..

[52] Kenny D.A., Kaniskan B., McCoach D.B. (2015). The Performance of RMSEA in Models with Small Degrees of Freedom. Sociol. Methods Res..

[53] Tucker L.R., Lewis C. (1973). A Reliability Coefficient for Maximum Likelihood Factor Analysis. Psychometrika.

[54] Korkmaz S., Goksuluk D., Zararsiz G. MVN: Multivariate Normality Tests. https://journal.r-project.org/archive/2014-2/korkmaz-goksuluk-zararsiz.pdf.

[55] Korkmaz S., Goksuluk D., Zararsiz G. (2014). MVN: An R Package for Assessing Multivariate Normality. R J..

[56] Thakkar J.J. (2020). Structural Equation Modelling: Application for Research and Practice (with AMOS and R).

[57] Zhao X., Lynch J.G., Chen Q. (2010). Reconsidering Baron and Kenny: Myths and Truths about Mediation Analysis. J. Consum. Res..

[58] Broekhof E., Bos M.G.N., Rieffe C. (2021). The Roles of Shame and Guilt in the Development of Aggression in Adolescents with and Without Hearing Loss. Res. Child Adolesc. Psychopathol..

[59] Carlo G., McGinley M., Davis A., Streit C. (2012). Behaving Badly or Goodly: Is It Because I Feel Guilty, Shameful, or Sympathetic? or Is It a Matter of What I Think?. New Dir. Youth Dev..

[60] Tangney J.P., Wagner P.E., Hill-Barlow D., Marschall D.E., Gramzow R. (1996). Relation of Shame and Guilt to Constructive versus Destructive Responses to Anger across the Lifespan. J. Personal. Soc. Psychol..

[61] Chang S.S.E., Jain S.P., Reimann M. (2021). The Role of Standards and Discrepancy Perfectionism in Maladaptive Consumption. J. Assoc. Consum. Res..

[62] Fedewa, Burns L.R., Gomez A.A. (2005). Positive and Negative Perfectionism and the Shame/Guilt Distinction: Adaptive and Maladaptive Characteristics. Personal. Individ. Differ..

[63] Stith S.M., Liu T., Davies L.C., Boykin E.L., Alder M.C., Harris J.M., Som A., McPherson M., Dees J.E.M.E.G. (2009). Risk Factors in Child Maltreatment: A Meta-Analytic Review of the Literature. Aggress. Violent Behav..

