# Supplementary material for: Does Parenting Perfectionism Ironically Increase Violent Behaviors from Parent towards Children?
Source: Children (Basel). 2023 Oct 19;10(10):1704. doi: 10.3390/children10101704 (PMC10605908; doi:10.3390/children10101704)
Supplement: Supplementary file 1 [file children-10-01704-s001.zip › children-2652696-supplementary.pdf]

Supplementary Materials

Figure S1. *Cross-Lag Model Investigating the Mediating Role of Parental Burnout Between Perfectionistic Strivings and Violence Towards the Offspring*

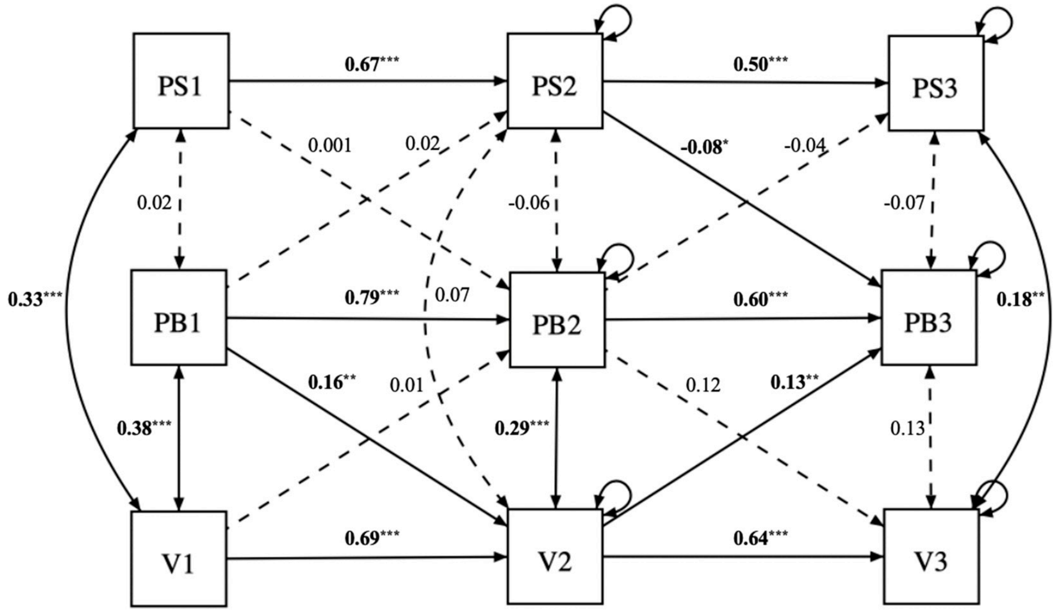

Note. PS1 = Perfectionistic Strivings at Time 1. PS2 = Perfectionistic Strivings at Time 2. PS3 = Perfectionistic Strivings at Time 3. PB1 = Parental Burnout at Time 1. PB2 = Parental Burnout at Time 2. PB3 = Parental Burnout at Time 3. V1 = Violence at Time 1. V2 = Violence at Time 2. V3 = Violence at Time 3. Significant paths and covariances are in black and their standardized coefficients are

marked in bold with asterisks ($^{*}p < .05$, $^{**}p < .01$, $^{***}p < .001$). Non-significant paths and covariances are depicted with dotted lines, and their coefficients are not in bold and with no asterisks. Bootstrapping = 1000 samples.

The first mediation model comprising perfectionistic strivings had an initial inadequate fit to the data: $\chi^2(15) = 162.52$, $p < .001$; CFI = .89; TLI = .73; RMSEA = 0.21, 95% CI = [0.18, 0.24], SRMR = .15. When covariances between highly correlated variables were added, the model showed better fit: $\chi^2(7) = 67.98$, $p < .001$; CFI = .95; TLI = .76; RMSEA = 0.19, 95% CI = [0.16, 0.24], SRMR = .04. Yet, as shown in **Figure S1** hereinabove, we did not find support for the hypothesized mediation. The results of the model did not change when controlling for social desirability.

Figure S2. *Cross-Lag Model Investigating the Mediating Role of Parental Burnout Between Perfectionistic Concerns and Violence Towards the Offspring*

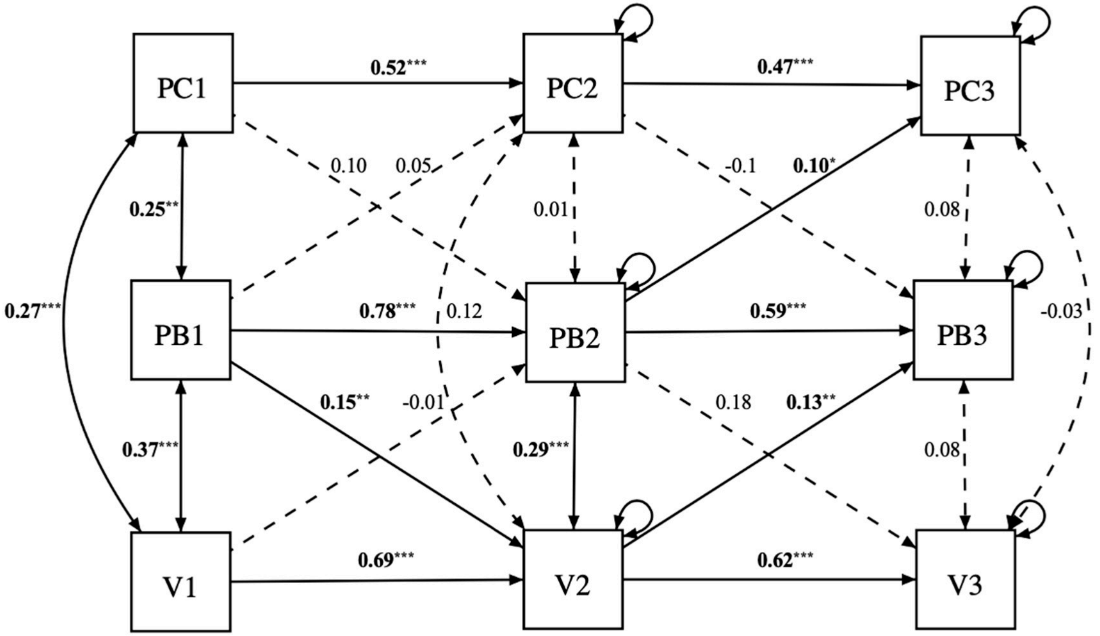

Note. PC1 = Perfectionistic Concerns at Time 1. PC2 = Perfectionistic Concerns at Time 2. PC3 = Perfectionistic Concerns at Time 3. PB1 = Parental Burnout at Time 1. PB2 = Parental Burnout at Time 2. PB3 = Parental Burnout at Time 3. V1 = Violence at Time 1. V2 = Violence at Time 2. V3 = Violence at Time 3. Significant paths and covariances are in black and their standardized coefficients are

marked in bold with asterisks ($^{*}p < .05$, $^{**}p < .01$, $^{***}p < .001$). Non-significant paths and covariances are depicted with dotted lines, and their coefficients are not in bold and with no asterisks. Bootstrapping = 1000 samples.

The second mediation model comprising perfectionistic concerns also had an initial bad fit to the data: $\chi^2(15) = 147.91$, $p < .001$; CFI = .89; TLI = .74; RMSEA = 0.20, 95% CI = [0.17, 0.23], SRMR = .12. After covariances were added, the final model had a better fit to the data: $\chi^2(4) = 57.33$, $p < .001$; CFI = .96; TLI = .60; RMSEA = 0.22, 95% CI = [0.18, 0.29], SRMR = .04. However, as shown in **Figure S2** hereinabove, we did not find support for the hypothesized mediation. These same results were observed when adding and controlling for social desirability.

Table S1. *Mediation Analyses between Perfectionistic Concerns, Parental Burnout, and Violence towards the Offspring, all at Time 1*

Effect	Standardized Estimate	*SE*	95% CI *LL*	95% CI *UL*	*Z*	*p*
Indirect (a*b)	0.04	0.004	0.002	0.02	2.48	.001**
PB1 → PC1	0.24	0.01	0.01	0.04	3.44	< .001***
PC1 → V1	0.18	0.13	0.15	0.66	3.19	.001**
Direct (c)	0.32	0.02	0.04	0.12	4.27	< .001***
Total (c + a*b)	0.37	0.02	0.06	0.13	5.13	< .001***

Note. PB1 = Parental Burnout at Time 1. PC1 = Perfectionistic Concerns at Time 1. V1 = Violence at Time 1. Bootstrapping = 1000 samples. $^{*}p < .05$. $^{**}p < .01$. $^{***}p < .001$.
